# Supplementary figures and images for: Discovering unknown Madagascar biodiversity: integrative taxonomy of raft spiders (Pisauridae: Dolomedes)
Source: PeerJ. 2024 Feb 27;12:e16781. doi: 10.7717/peerj.16781 (PMC10906265; doi:10.7717/peerj.16781)

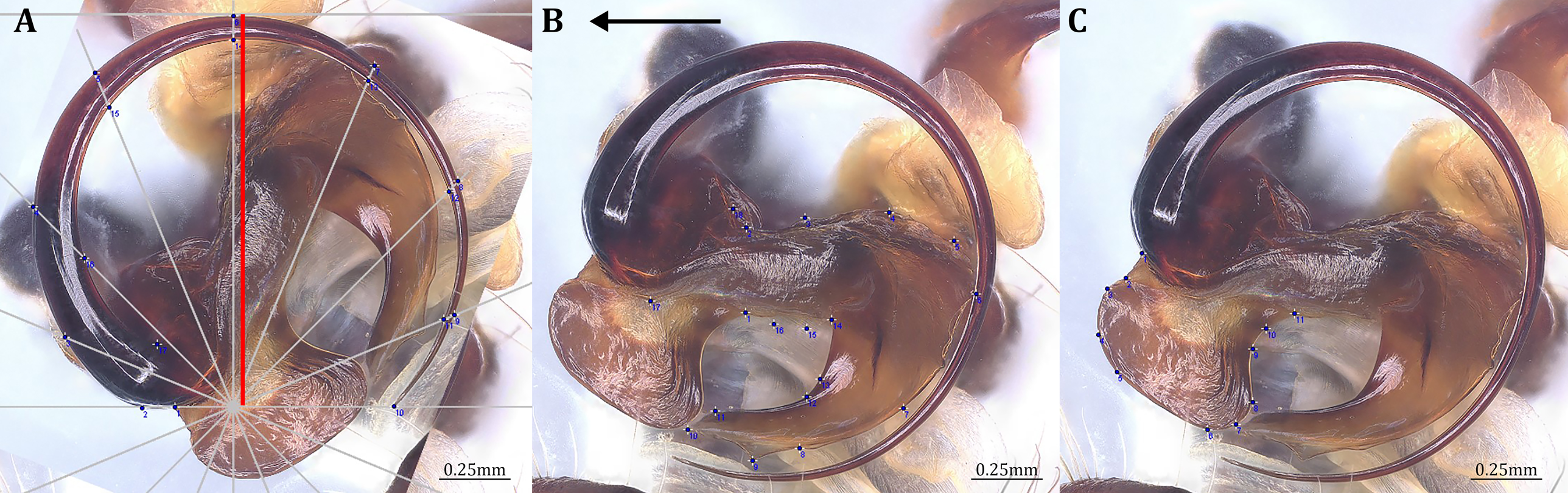

Supplement: Supplemental Information 1 — (A) Embolus, the gray lines represent the reference lines for landmarks plotting, the red line represent the diameter of embolic ring (De). (B) Fulcrum. (C) Lateral subterminal apophysis. The black arrow showing the dorsal direction. [file peerj-12-16781-s001.jpg]

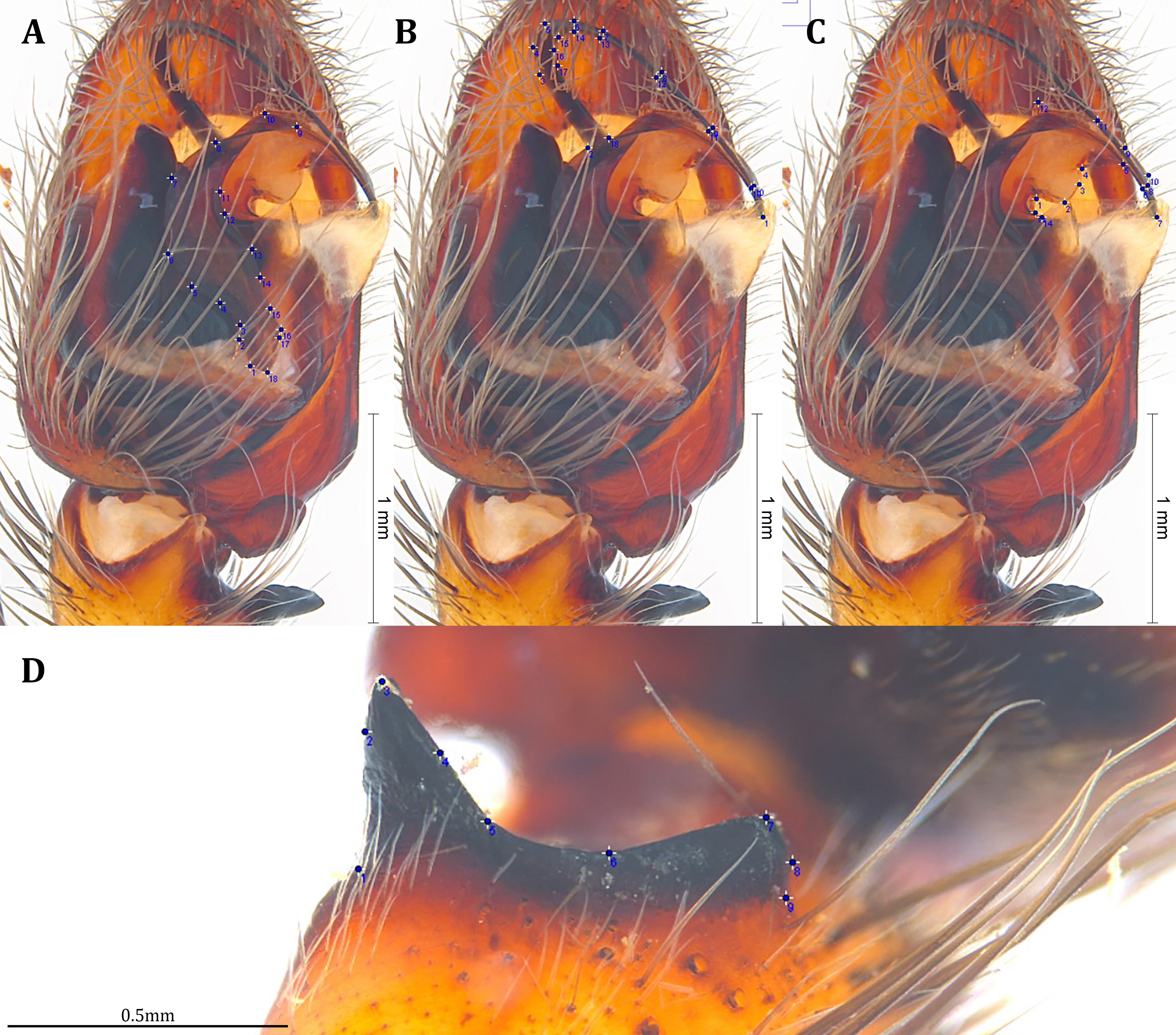

Supplement: Supplemental Information 2 — (A) Median apophysis, ventral view. (B) Embolus, ventral view. (C) Fulcrum, dorsal view. (D) retrolatateral tibial apophysis, posterolateral view. [file peerj-12-16781-s002.jpg]

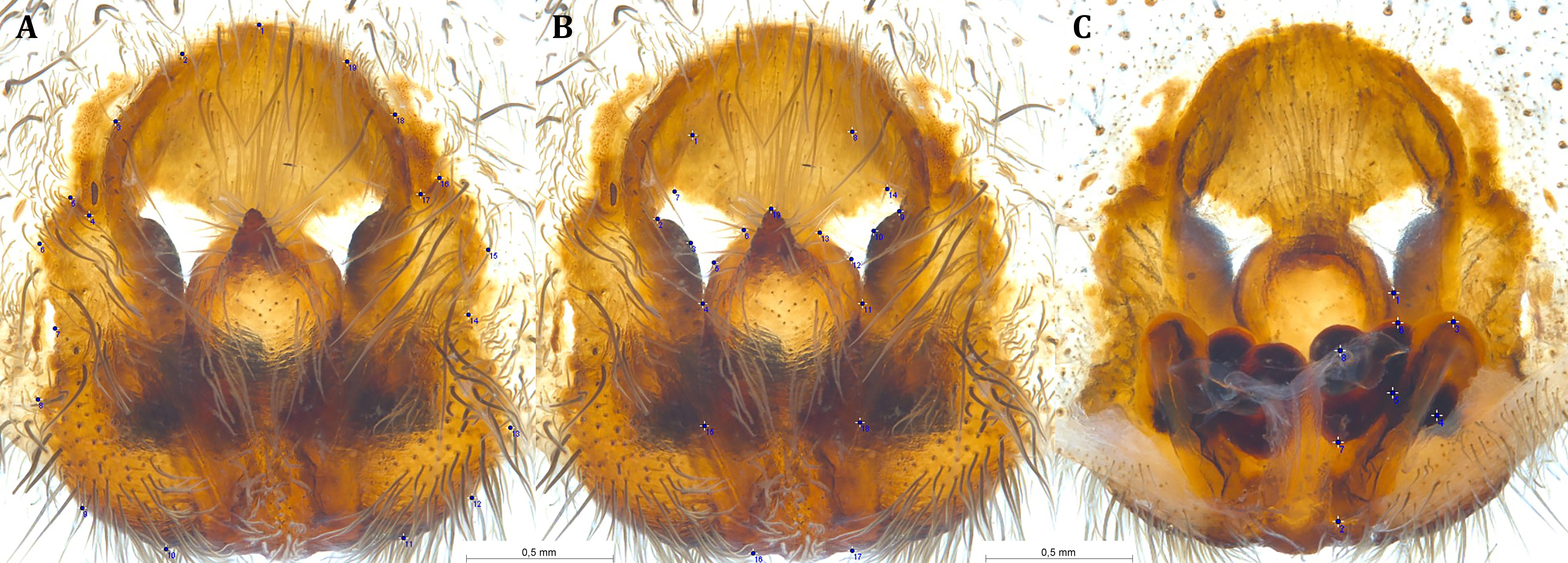

Supplement: Supplemental Information 3 — A) Epigynal margin, ventral view. (B) Epigynal middle field, ventral view. (C) Vulva arrangement, dorsal view. [file peerj-12-16781-s003.jpg]

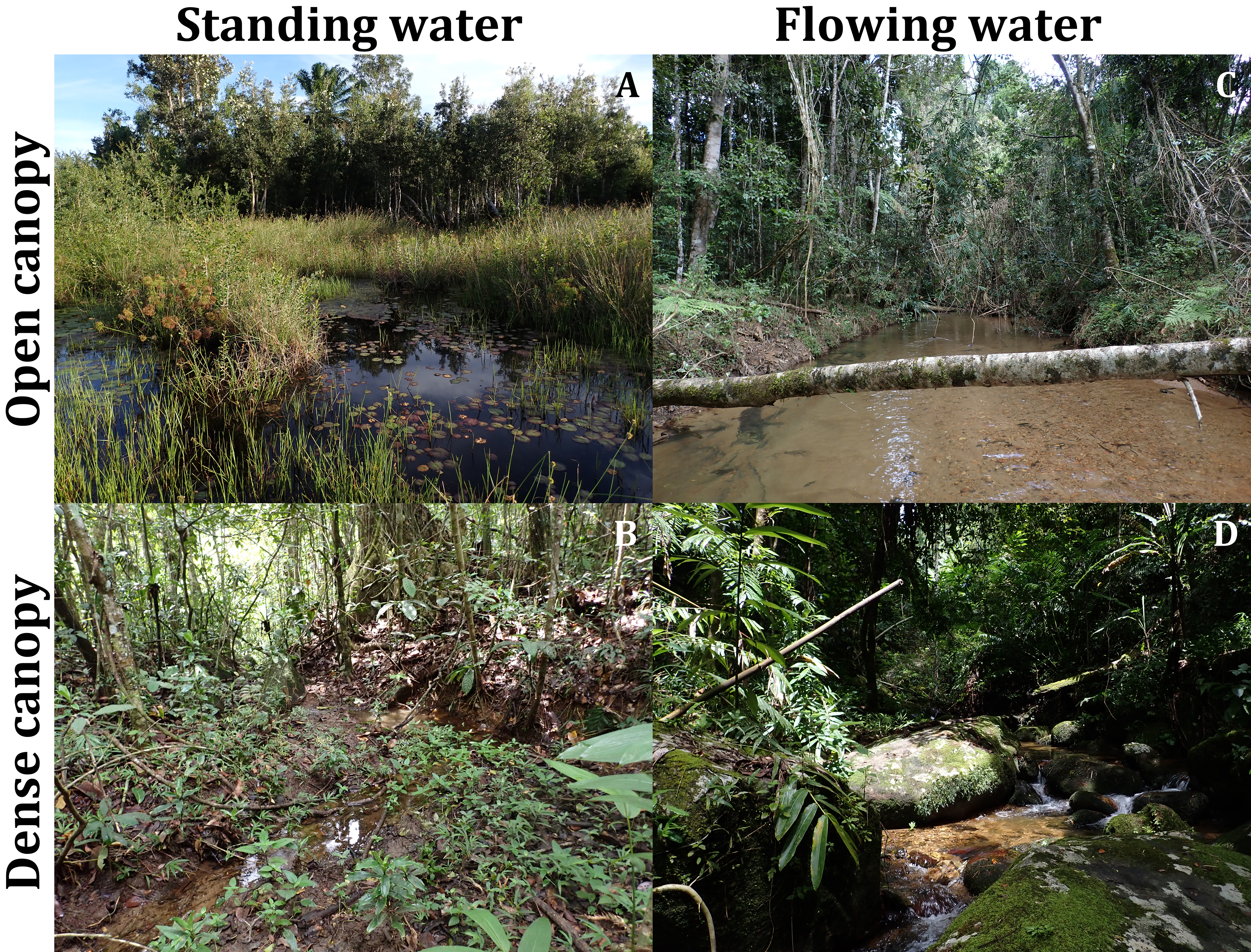

Supplement: Supplemental Information 4 — (A) The pond at the countryside of Sambava, represents standing water with an open canopy. (B) Understory swamps at Parc National de Marojejy, represent standing water with dense canopy. (C) The river along Circuit Tsakoka, Parc National d’Andasibe-Mantadia, represents flowing water with an open canopy. (D) The stream at Parc National de Marojejy, represents flowing water with a dense canopy. [file peerj-12-16781-s004.jpg]

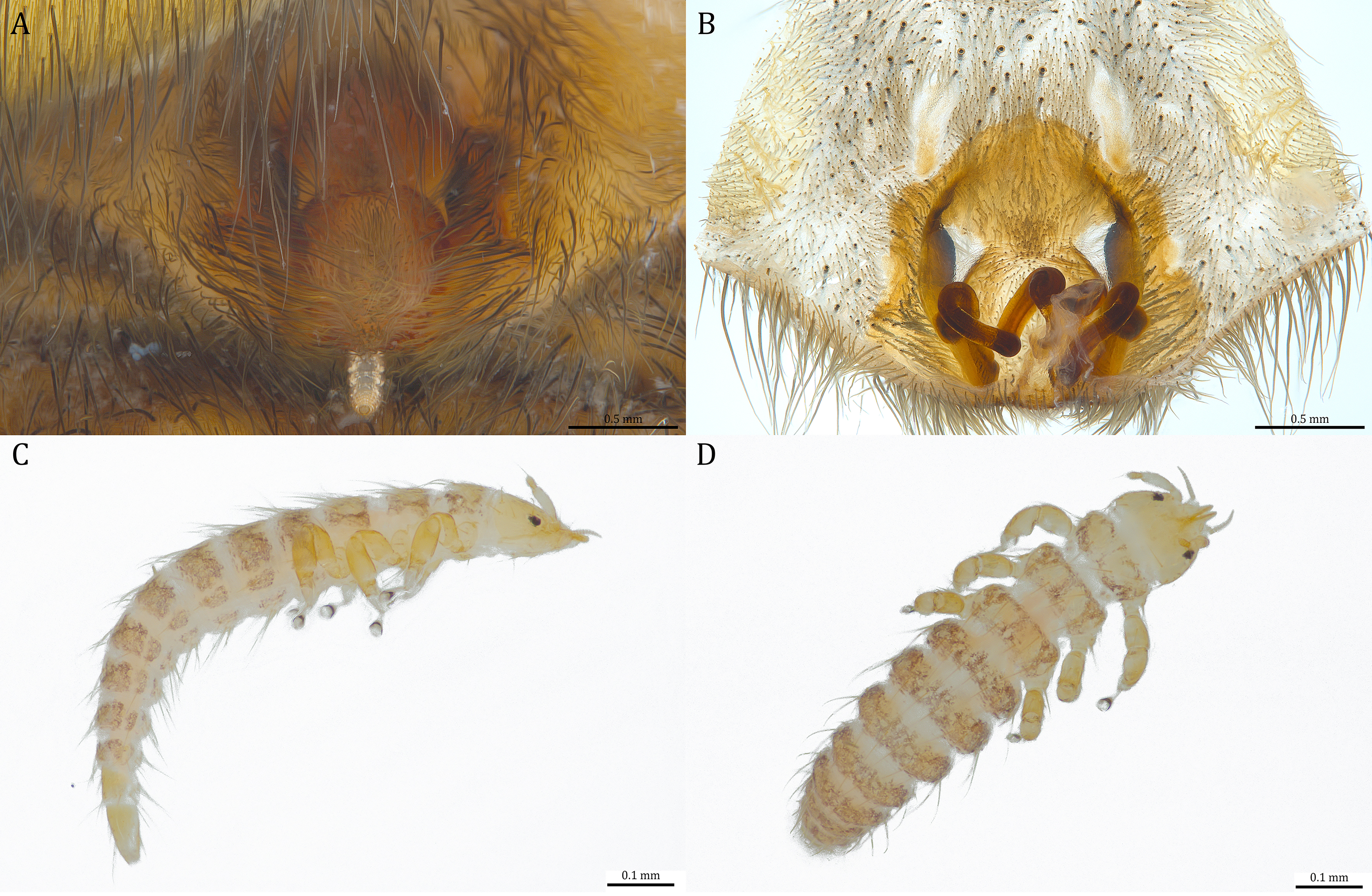

Supplement: Supplemental Information 5 — (A–B) Parasitized epigynum: (A) Ventral view; (B) dorsal view, showing the deformed vulva. (C–D) The Mantispidae larva: (C) Dorsal view; (D) lateral view. [file peerj-12-16781-s005.jpg]
