# Supplementary material for: Discovering unknown Madagascar biodiversity: integrative taxonomy of raft spiders (Pisauridae: Dolomedes)
Source: PeerJ. 2024 Feb 27;12:e16781. doi: 10.7717/peerj.16781 (PMC10906265; doi:10.7717/peerj.16781)
Supplement: Supplemental Information 6 — RTA: retrolateral tibial apophysis, LA: lateral subterminal apophysis. [file peerj-12-16781-s006.docx]

**Table S1:
Descriptions of the landmarks selected within each structure.**
RTA: retrolateral tibial apophysis, LA: lateral subterminal apophysis.

| **Structure** | **View** | **Landmark** | **Type of landmark(s)** | **Description** |
| --- | --- | --- | --- | --- |
| Epigynal margin | Ventral | 1–8, 13–19 | II | Points of maximum inflection along the epigynum margin |
|  |  | 9, 12 | II | Points of intersection between the epigynum and the epigastric furrow |
|  |  | 10, 11 | II | Lowest points of the epigynal lateral lobes |
| Epigynal middle field | Ventral | 1, 8 | II | Starting points of the epigynal folds |
|  |  | 2, 3, 5, 7, 9, 10, 12, 14 | II | Points of maximum inflection along the window of the epigynal middle field |
|  |  | 4, 11 | II | Copulatory openings |
|  |  | 6, 13 | II | The narrowest part between two windows of the epigynal middle field |
|  |  | 15, 18 | II | The widest part of the epigynal middle field |
|  |  | 16, 17 | II | The ending points of the epigynal folds |
|  |  | 19 | II | The highest extension point of the lower epigynal middle field |
| Vulva arrangement | Dorsal | 1 | II | Copulatory opening |
|  |  | 2, 3, 6, 7 | II | Major curve points of the vulva |
|  |  | 4 | II | Ending point of the copulatory duct |
|  |  | 5 | II | Starting point of the fertilization duct |
|  |  | 8 | II | The point where the flake part of the fertilization duct started |
| Left median apophysis | Ventral | 1, 18 | II | Intersection points between the sclerotized part of the median apophysis and the tegulum |
|  |  | 2, 17 | II | Points where the membranous part of the median apophysis ended |
|  |  | 3–16 | II | Points of maximum inflection along the edge of the median apophysis |
| Left embolus | Ventral | 1 | II | Tip of the embolus |
|  |  | 2, 18 | II | Base of the embolus |
|  |  | 3, 17 | II | Points where the embolus start curving |
|  |  | 4, 5, 6,7, 8, 10–16 | II | Points of maximum inflection along the edge of the embolus |
| Left fulcrum | Ventral | 1–6, 8–13 | II | Points of maximum inflection along the edge of the fulcrum |
|  |  | 7 | II | Tip of the fulcrum |
|  |  | 14 | II | Base of the fulcrum |
| RTA | Posterolateral | 1, 9 | II | Base of the RTA |
|  |  | 3 | II | Tip of the ventral lobe of the RTA |
|  |  | 7 | II | Tip of the dorsal lobe of the RTA |
|  |  | 2, 4–6, 8 | II | Points of maximum inflection of the RTA edge |
| Right embolus, expanded | Retrolateral | 1 | II | Intersection point between the embolus and the lateral subterminal apophysis |
|  |  | 2 | II | Point of maximum inflection of the outer edge of the embolus |
|  |  | 3–9, 11–15 | Semi | Intersection points between the edge of the embolus and the reference line |
|  |  | 10 | II | Tip of the embolus |
|  |  | 16 | II/Semi | Intersection point between the inner edge of the embolus and the reference line; in the case that the edge and the line does not intersect, the point represent the maximum inflection of the inner edge of the embolus |
|  |  | 17 | II | Base of the inner edge of the embolus |
| Right fulcrum, expanded | Retrolateral | 1 | II | Intersection point between the post edge of the fulcrum and the ventral edge of the LA |
|  |  | 2–9, 11–18 | II | Points of maximum inflection along the edge of the fulcrum |
|  |  | 10 | II | Tip of the fulcrum |
|  |  | 17, 18 | II | Base of the fulcrum |
| LA, expanded | Retrolateral | 1 | II | Intersection point between the embolus and the LA |
|  |  | 2–10 | II | Points of maximum inflection along the edge of the LA |
|  |  | 11 | II | Intersection point between the post edge of the fulcrum and the ventral edge of the LA |
