# Supplementary material for: Discovering unknown Madagascar biodiversity: integrative taxonomy of raft spiders (Pisauridae: Dolomedes)
Source: PeerJ. 2024 Feb 27;12:e16781. doi: 10.7717/peerj.16781 (PMC10906265; doi:10.7717/peerj.16781)
Supplement: Supplemental Information 7 [file peerj-12-16781-s007.docx]

**Table S2:**
**GenBank accession numbers of the COI sequences included in this study.**

| Species | Voucher | Genbank accession number | Note |
| --- | --- | --- | --- |
| *Dolomedes kalanoro* | KPARA00186 | OR284697 | juvenile |
| *Dolomedes kalanoro* | KPARA00187 | OR284698 | juvenile |
| *Dolomedes kalanoro* | KPARA00195 | OR284699 | juvenile |
| *Dolomedes kalanoro* | KPARA00196 | OR284700 | juvenile |
| *Dolomedes kalanoro* | KPARA00212 | OR284701 | juvenile |
| *Dolomedes kalanoro* | KPARA00213 | OR284702 | juvenile |
| *Dolomedes bedjanic* sp. nov. | KPARA00226 | OR284703 | juvenile |
| *Dolomedes kalanoro* | KPARA00214 | OR284704 | juvenile |
| *Dolomedes kalanoro* | KPARA00184 | OR284705 |  |
| *Dolomedes kalanoro* | KPARA00185 | OR284706 |  |
| *Dolomedes kalanoro* | KPARA00193 | OR284707 |  |
| *Dolomedes kalanoro* | KPARA00201 | OR284708 |  |
| *Dolomedes kalanoro* | KPARA00228 | OR284709 |  |
| *Dolomedes kalanoro* | KPARA00231 | OR284710 |  |
| *Dolomedes kalanoro* | KPARA00207 | OR284711 |  |
| *Dolomedes kalanoro* | KPARA00208 | OR284712 |  |
| *Dolomedes raptor* | ABARA00394 | OR284713 |  |
| *Dolomedes* madsp1 | KPARA00215 | OR284714 | Cazanovae, in prep. |
| *Dolomedes* madsp1 | KPARA00216 | OR284715 | Cazanovae, in prep. |
| *Dolomedes* madsp1 | KPARA00217 | OR284716 | Cazanovae, in prep. |
| *Dolomedes* madsp1 | KPARA00225 | OR284717 | Cazanovae, in prep. |
| *Dolomedes* madsp1 | KPARA00172 | OR284718 | Cazanovae, in prep. |
| *Dolomedes* madsp1 | KPARA00173 | OR284719 | Cazanovae, in prep. |
| *Dolomedes* madsp1 | KPARA00174 | OR284720 | Cazanovae, in prep. |
| *Dolomedes* madsp1 | KPARA00175 | OR284721 | Cazanovae, in prep. |
| *Dolomedes* madsp1 | KPARA00211 | OR284722 | Cazanovae, in prep. |
| *Dolomedes* madsp1 | KPARA00246 | OR284723 | Cazanovae, in prep. |
| *Dolomedes* madsp1 | KPARA00408 | OR284724 | Cazanovae, in prep. |
| *Dolomedes bedjanic* sp. nov. | KPARA00192 | OR284725 |  |
| *Dolomedes bedjanic* sp. nov. | KPARA00194 | OR284726 |  |
| *Dolomedes bedjanic* sp. nov. | KPARA00227 | OR284727 |  |
| *Dolomedes bedjanic* sp. nov. | KPARA00232 | OR284728 |  |
| *Dolomedes bedjanic* sp. nov. | KPARA00233 | OR284729 |  |
| *Dolomedes bedjanic* sp. nov. | KPARA00202 | OR284730 |  |
| *Dolomedes bedjanic* sp. nov. | KPARA00247 | OR284731 |  |
| *Dolomedes bedjanic* sp. nov. | KPARA00251 | OR284732 |  |
| *Dolomedes bedjanic* sp. nov. | KPARA00252 | OR284733 |  |
| *Dolomedes bedjanic* sp. nov. | KPARA00129 | OR284734 |  |
| *Dolomedes bedjanic* sp. nov. | KPARA00130 | OR284735 |  |
| *Dolomedes bedjanic* sp. nov. | KPARA00144 | OR284736 |  |
| *Dolomedes bedjanic* sp. nov. | KPARA00153 | OR284737 |  |
| *Dolomedes bedjanic* sp. nov. | KPARA00166 | OR284738 |  |
| *Dolomedes bedjanic* sp. nov. | KPARA00234 | OR284739 |  |
| *Dolomedes bedjanic* sp. nov. | KPARA00235 | OR284740 |  |
| *Dolomedes gregoric* sp. nov. | KPARA00248 | OR284741 |  |
| *Dolomedes gregoric* sp. nov. | KPARA00249 | OR284742 |  |
| *Dolomedes gregoric* sp. nov. | KPARA00250 | OR284743 |  |
| *Dolomedes gregoric* sp. nov. | KPARA00253 | OR284744 |  |
| *Dolomedes gregoric* sp. nov. | KPARA00254 | OR284745 |  |
| *Dolomedes hydatostellla* sp. nov. | KPARA00157 | OR284746 |  |
| *Dolomedes hydatostellla* sp. nov. | KPARA00158 | OR284747 |  |
| *Dolomedes hydatostellla* sp. nov. | KPARA00163 | OR284748 |  |
| *Dolomedes hydatostellla* sp. nov. | KPARA00258 | OR284749 |  |
| *Dolomedes rotundus* sp. nov. | KPARA00238 | OR284750 |  |
| *Dolomedes rotundus* sp. nov. | KPARA00239 | OR284751 |  |
| *Dolomedes rotundus* sp. nov. | KPARA00240 | OR284752 |  |
| *Dolomedes rotundus* sp. nov. | KPARA00242 | OR284753 |  |
| *Dolomedes rotundus* sp. nov. | KPARA00243 | OR284754 |  |
