# Supplementary material for: Discovering unknown Madagascar biodiversity: integrative taxonomy of raft spiders (Pisauridae: Dolomedes)
Source: PeerJ. 2024 Feb 27;12:e16781. doi: 10.7717/peerj.16781 (PMC10906265; doi:10.7717/peerj.16781)
Supplement: Supplemental Information 8 — De: diameter of embolic ring; Df: degrees of freedom; SS: sum of square; MS: mean square; significance threshold: p-value < 0.05 [file peerj-12-16781-s008.docx]

**Table S3:
Results of the one-way analyses of variance.**
Measurements of the six selected structures are significantly different among the five morphospecies in both sexes. D_e_: diameter of embolic ring; Df: degrees of freedom; SS: sum of square; MS: mean square; significance threshold: p-value < 0.05

| **Structure** | **Factor** | **Df** | **SS** | **MS** | **F** | ***p*** |
| --- | --- | --- | --- | --- | --- | --- |
| Female carapace width | Species | 4 | 49.82 | 12.455 | 22.78 | <0.001 |
|  | Residual | 24 | 13.12 | 0.547 |  |  |
| Male carapace width | Species | 4 | 20.833 | 5.208 | 25.31 | <0.001 |
|  | Residual | 17 | 3.498 | 0.206 |  |  |
| Female leg I length / carapace width | Species | 4 | 8.883 | 2.221 | 92.43 | <0.001 |
|  | Residual | 24 | 0.577 | 0.024 |  |  |
| Male leg I length / carapace width | Species | 4 | 10.437 | 2.6092 | 52.6 | <0.001 |
|  | Residual | 17 | 0.843 | 0.0496 |  |  |
| Female tarsus I length / leg I length | Species | 4 | 0.00267 | 0.00067 | 90.33 | <0.001 |
|  | Residual | 24 | 0.00018 | 0.00007 |  |  |
| Male tarsus I length / leg I length | Species | 4 | 0.00168 | 0.00042 | 12.34 | <0.001 |
|  | Residual | 17 | 0.00058 | 0.00003 |  |  |
| Female palp length / carapace width | Species | 4 | 0.3721 | 0.09302 | 33.32 | <0.001 |
|  | Residual | 24 | 0.067 | 0.00279 |  |  |
| Male palp length / carapace width | Species | 4 | 0.805 | 0.20125 | 57.25 | <0.001 |
|  | Residual | 17 | 0.0598 | 0.00352 |  |  |
| Male cymbium length / palp tibia length | Species | 4 | 0.4816 | 0.12039 | 66.24 | <0.001 |
|  | Residual | 17 | 0.0309 | 0.00182 |  |  |
| Male D_e_ | Species | 4 | 1.8806 | 0.4702 | 605.4 | <0.001 |
|  | Residual | 17 | 0.0132 | 0.0008 |  |  |
