# Supplementary material for: Discovering unknown Madagascar biodiversity: integrative taxonomy of raft spiders (Pisauridae: Dolomedes)
Source: PeerJ. 2024 Feb 27;12:e16781. doi: 10.7717/peerj.16781 (PMC10906265; doi:10.7717/peerj.16781)
Supplement: Supplemental Information 9 — De: diameter of embolic ring; diff: difference between means of the two groups; lwr: lower end point of 95% confident interval; upr: upper end point of 95% confident interval; significance threshold: p-value < 0.05; significant p-value were in bold. [file peerj-12-16781-s009.docx]

**Table S4:
Pairwise comparisons in measurements of the six selected structures between all pair combinations of the five morphospecies.**
D_e_: diameter of embolic ring; diff: difference between means of the two groups; lwr: lower end point of 95% confident interval; upr: upper end point of 95% confident interval; significance threshold: p-value < 0.05; significant p-value were in bold.

| **Structure** | **Combinations** | **Female** | | | | **Male** | | | |
| --- | --- | --- | --- | --- | --- | --- | --- | --- | --- |
|  |  | diff | lwr | upr | *p* | diff | lwr | upr | *p* |
| Carapace width | gregoric - kalanoro | -1.468 | -3.131 | 0.196 | 0.102 | -0.703 | -1.858 | 0.452 | 0.378 |
|  | bedjanic - kalanoro | 0.195 | -1.040 | 1.430 | 0.990 | -0.191 | -0.999 | 0.617 | 0.949 |
|  | hydatostella - kalanoro | -2.714 | -4.175 | -1.252 | **<0.001** | -1.818 | -2.973 | -0.663 | **0.001** |
|  | rotundus - kalanoro | -2.944 | -4.608 | -1.280 | **<0.001** | -2.285 | -3.120 | -1.449 | **<0.001** |
|  | bedjanic - gregoric | 1.662 | 0.276 | 3.048 | **0.013** | 0.512 | -0.594 | 1.619 | 0.631 |
|  | hydatostella - gregoric | -1.246 | -2.837 | 0.345 | 0.177 | -1.115 | -2.495 | 0.265 | 0.147 |
|  | rotundus - gregoric | -1.477 | -3.255 | 0.302 | 0.137 | -1.582 | -2.708 | -0.455 | **0.004** |
|  | hydatostella - bedjanic | -2.908 | -4.043 | -1.773 | **<0.001** | -1.627 | -2.734 | -0.521 | **0.003** |
|  | rotundus - bedjnaic | -3.139 | -4.525 | -1.753 | **<0.001** | -2.094 | -2.862 | -1.326 | **<0.001** |
|  | rotundus - hydatostella | -0.231 | -1.822 | 1.360 | 0.993 | -0.467 | -1.593 | 0.660 | 0.718 |
| Leg I length / carapace width | gregoric - kalanoro | 0.250 | -0.099 | 0.599 | 0.248 | 0.338 | -0.229 | 0.905 | 0.397 |
|  | bedjanic - kalanoro | 0.039 | -0.220 | 0.298 | 0.992 | 0.114 | -0.283 | 0.511 | 0.902 |
|  | hydatostella - kalanoro | -1.134 | -1.440 | -0.827 | **<0.001** | -1.333 | -1.900 | -0.766 | **<0.001** |
|  | rotundus - kalanoro | -1.221 | -1.570 | -0.873 | **<0.001** | -1.309 | -1.719 | -0.899 | **<0.001** |
|  | bedjanic - gregoric | -0.211 | -0.502 | 0.079 | 0.236 | -0.224 | -0.767 | 0.319 | 0.721 |
|  | hydatostella - gregoric | -1.384 | -1.717 | -1.050 | **<0.001** | -1.671 | -2.349 | -0.994 | **<0.001** |
|  | rotundus - gregoric | -1.471 | -1.844 | -1.099 | **<0.001** | -1.647 | -2.201 | -1.094 | **<0.001** |
|  | hydatostella - bedjanic | -1.173 | -1.411 | -0.935 | **<0.001** | -1.447 | -1.990 | -0.904 | **<0.001** |
|  | rotundus - bedjanic | -1.260 | -1.551 | -0.970 | **<0.001** | -1.423 | -1.800 | -1.046 | **<0.001** |
|  | rotundus - hydatostella | -0.088 | -0.421 | 0.246 | 0.935 | 0.024 | -0.529 | 0.577 | 1.000 |
| Tarsus I length / leg I length | gregoric - kalanoro | 0.002 | -0.005 | 0.008 | 0.936 | 0.003 | -0.012 | 0.018 | 0.964 |
|  | bedjanic - kalanoro | 0.000 | -0.004 | 0.005 | 1.000 | -0.004 | -0.014 | 0.007 | 0.801 |
|  | hydatostella - kalanoro | -0.022 | -0.028 | -0.017 | **<0.001** | -0.020 | -0.035 | -0.005 | **0.006** |
|  | rotundus - kalanoro | -0.019 | -0.025 | -0.012 | **<0.001** | -0.019 | -0.030 | -0.008 | **<0.001** |
|  | bedjanic - gregoric | -0.001 | -0.006 | 0.004 | 0.926 | -0.007 | -0.021 | 0.007 | 0.584 |
|  | hydatostella - gregoric | -0.024 | -0.030 | -0.018 | **<0.001** | -0.023 | -0.041 | -0.005 | **0.008** |
|  | rotundus - gregoric | -0.020 | -0.027 | -0.014 | **<0.001** | -0.022 | -0.037 | -0.008 | **0.002** |
|  | hydatostella - bedjanic | -0.023 | -0.027 | -0.018 | **<0.001** | -0.016 | -0.030 | -0.002 | **0.023** |
|  | rotundus - bedjanic | -0.019 | -0.024 | -0.014 | **<0.001** | -0.015 | -0.025 | -0.005 | **0.002** |
|  | rotundus - hydatostella | 0.004 | -0.002 | 0.010 | 0.324 | 0.001 | -0.013 | 0.016 | 0.999 |
| Palp length / carapace width | gregoric - kalanoro | 0.054 | -0.065 | 0.173 | 0.668 | 0.078 | -0.073 | 0.228 | 0.539 |
|  | bedjanic - kalanoro | 0.049 | -0.040 | 0.137 | 0.497 | 0.211 | 0.105 | 0.316 | **<0.001** |
|  | hydatostella - kalanoro | -0.201 | -0.305 | -0.096 | **<0.001** | -0.308 | -0.459 | -0.157 | **<0.001** |
|  | rotundus - kalanoro | -0.226 | -0.345 | -0.107 | **<0.001** | -0.223 | -0.332 | -0.113 | **<0.001** |
|  | bedjanic - gregoric | -0.005 | -0.105 | 0.094 | 1.000 | 0.133 | -0.011 | 0.278 | 0.079 |
|  | hydatostella - gregoric | -0.255 | -0.368 | -0.141 | **<0.001** | -0.385 | -0.566 | -0.205 | **<0.001** |
|  | rotundus - gregoric | -0.280 | -0.407 | -0.153 | **<0.001** | -0.300 | -0.447 | -0.153 | **<0.001** |
|  | hydatostella - bedjanic | -0.249 | -0.330 | -0.168 | **<0.001** | -0.518 | -0.663 | -0.374 | **<0.001** |
|  | rotundus - bedjanic | -0.275 | -0.374 | -0.176 | **<0.001** | -0.433 | -0.534 | -0.333 | **<0.001** |
|  | rotundus - hydatostella | -0.025 | -0.139 | 0.088 | 0.964 | 0.085 | -0.062 | 0.232 | 0.428 |
| Structure | Level combinations | Male | | | |  |  |  |  |
|  |  | diff | lwr | upr | *p* |  |  |  |  |
| Cymbium length / palp tibia length | gregoric - kalanoro | -0.132 | -0.240 | -0.023 | **0.013** |  |  |  |  |
|  | bedjanic - kalanoro | -0.366 | -0.442 | -0.290 | **<0.001** |  |  |  |  |
|  | hydatostella - kalanoro | -0.040 | -0.149 | 0.068 | 0.788 |  |  |  |  |
|  | rotundus - kalanoro | -0.093 | -0.171 | -0.014 | 0.017 |  |  |  |  |
|  | bedjanic - gregoric | -0.234 | -0.338 | -0.130 | **<0.001** |  |  |  |  |
|  | hydatostella - gregoric | 0.092 | -0.038 | 0.221 | 0.246 |  |  |  |  |
|  | rotundus - gregoric | 0.039 | -0.067 | 0.145 | 0.790 |  |  |  |  |
|  | hydatostella - bedjanic | 0.325 | 0.221 | 0.429 | **<0.001** |  |  |  |  |
|  | rotundus - bedjanic | 0.273 | 0.201 | 0.345 | **<0.001** |  |  |  |  |
|  | rotundus - hydatostella | -0.052 | -0.158 | 0.054 | 0.574 |  |  |  |  |
| D_e_ | gregoric - kalanoro | -0.256 | -0.327 | -0.185 | **<0.001** |  |  |  |  |
|  | bedjanic - kalanoro | -0.446 | -0.496 | -0.396 | **<0.001** |  |  |  |  |
|  | hydatostella - kalanoro | -0.786 | -0.857 | -0.715 | **<0.001** |  |  |  |  |
|  | rotundus - kalanoro | -0.758 | -0.809 | -0.706 | **<0.001** |  |  |  |  |
|  | bedjanic - gregoric | -0.190 | -0.258 | -0.122 | **<0.001** |  |  |  |  |
|  | hydatostella - gregoric | -0.530 | -0.615 | -0.445 | **<0.001** |  |  |  |  |
|  | rotundus - gregoric | -0.502 | -0.571 | -0.432 | **<0.001** |  |  |  |  |
|  | hydatostella - bedjanic | -0.340 | -0.408 | -0.272 | **<0.001** |  |  |  |  |
|  | rotundus - bedjanic | -0.312 | -0.359 | -0.264 | **<0.001** |  |  |  |  |
|  | rotundus - hydatostella | 0.028 | -0.041 | 0.098 | 0.726 |  |  |  |  |
