# Supplementary material for: Discovering unknown Madagascar biodiversity: integrative taxonomy of raft spiders (Pisauridae: Dolomedes)
Source: PeerJ. 2024 Feb 27;12:e16781. doi: 10.7717/peerj.16781 (PMC10906265; doi:10.7717/peerj.16781)
Supplement: Supplemental Information 10 — RTA: retrolateral tibial apophysis, LA: lateral subterminal apophysis. [file peerj-12-16781-s010.docx]

**Table S5:
Proportion and cumulative variances in the shape components of each selected structure explained by the first five principal component axes (PC).**
RTA: retrolateral tibial apophysis, LA: lateral subterminal apophysis.

| **Structure** | **View** | **Group** | **Type of explained variance** | **PC1** | **PC2** | **PC3** | **PC4** | **PC5** |
| --- | --- | --- | --- | --- | --- | --- | --- | --- |
| Epigynal margin | Ventral | kalanoro | Proportion | 40.47 | 14.00 | 8.50 | 7.77 | 6.28 |
|  |  |  | Cumulative | 40.47 | 54.47 | 62.97 | 70.74 | 77.02 |
|  |  | hydatostella | Proportion | 34.29 | 24.70 | 10.89 | 10.37 | 8.74 |
|  |  |  | Cumulative | 34.29 | 58.99 | 69.88 | 80.25 | 88.99 |
| Epigynal middle field | Ventral | kalanoro | Proportion | 53.11 | 18.74 | 7.83 | 6.32 | 3.59 |
|  |  |  | Cumulative | 53.11 | 71.85 | 79.68 | 86.00 | 89.59 |
|  |  | hydatostella | Proportion | 64.85 | 17.64 | 7.11 | 4.34 | 3.09 |
|  |  |  | Cumulative | 64.85 | 82.49 | 89.60 | 93.94 | 97.03 |
| Vulva arrangement | Dorsal | kalanoro | Proportion | 79.61 | 7.15 | 4.67 | 2.44 | 2.30 |
|  |  |  | Cumulative | 79.61 | 86.76 | 91.43 | 93.87 | 96.17 |
|  |  | hydatostella | Proportion | 61.21 | 19.59 | 7.39 | 4.96 | 4.46 |
|  |  |  | Cumulative | 61.21 | 80.80 | 88.19 | 93.15 | 97.61 |
| Left median apophysis | Ventral | kalanoro | Proportion | 44.40 | 20.38 | 8.50 | 7.30 | 5.59 |
|  |  |  | Cumulative | 44.40 | 64.78 | 73.28 | 80.58 | 86.17 |
|  |  | hydatostella | Proportion | 36.39 | 22.09 | 15.21 | 12.31 | 7.49 |
|  |  |  | Cumulative | 36.39 | 58.48 | 73.69 | 86.00 | 93.49 |
| Left embolus | Ventral | kalanoro | Proportion | 59.32 | 20.75 | 8.41 | 3.94 | 2.56 |
|  |  |  | Cumulative | 59.32 | 80.07 | 88.48 | 92.42 | 94.98 |
|  |  | hydatostella | Proportion | 62.50 | 13.29 | 8.15 | 7.11 | 4.36 |
|  |  |  | Cumulative | 62.50 | 75.79 | 83.94 | 91.05 | 95.41 |
| Left fulcrum | Ventral | kalanoro | Proportion | 52.60 | 17.08 | 9.73 | 7.22 | 3.97 |
|  |  |  | Cumulative | 52.60 | 69.68 | 79.41 | 86.63 | 90.60 |
|  |  | hydatostella | Proportion | 45.84 | 23.15 | 13.75 | 8.49 | 5.32 |
|  |  |  | Cumulative | 45.84 | 68.99 | 82.74 | 91.23 | 96.55 |
| RTA | Posterolateral | kalanoro | Proportion | 39.49 | 30.53 | 10.77 | 7.59 | 4.06 |
|  |  |  | Cumulative | 39.49 | 70.02 | 80.79 | 88.38 | 92.44 |
|  |  | hydatostella | Proportion | 55.74 | 17.43 | 14.34 | 6.43 | 3.14 |
|  |  |  | Cumulative | 55.74 | 73.17 | 87.51 | 93.94 | 97.08 |
| Right embolus, expanded | Retrolateral | kalanoro | Proportion | 84.00 | 9.56 | 3.32 | 1.33 | 0.71 |
|  |  |  | Cumulative | 84.00 | 93.56 | 96.88 | 98.21 | 98.92 |
|  |  | hydatostella | Proportion | 65.39 | 15.85 | 7.03 | 6.89 | 3.45 |
|  |  |  | Cumulative | 65.39 | 81.24 | 88.27 | 95.16 | 98.61 |
| Right fulcrum, expanded | Retrolateral | kalanoro | Proportion | 30.81 | 21.05 | 14.43 | 9.94 | 6.94 |
|  |  |  | Cumulative | 30.81 | 51.86 | 66.29 | 76.23 | 83.17 |
|  |  | hydatostella | Proportion | 39.21 | 23.17 | 15.43 | 10.19 | 5.92 |
|  |  |  | Cumulative | 39.21 | 62.38 | 77.81 | 88.00 | 93.92 |
| LA, expanded | Retrolateral | kalanoro | Proportion | 36.59 | 22.35 | 16.53 | 8.57 | 6.90 |
|  |  |  | Cumulative | 36.59 | 58.94 | 75.46 | 84.03 | 90.93 |
|  |  | hydatostella | Proportion | 41.47 | 24.44 | 15.46 | 8.89 | 5.64 |
|  |  |  | Cumulative | 41.47 | 65.91 | 81.37 | 90.26 | 95.89 |
